# Levels of Par-1 kinase determine the localization of Bruchpilot at the *Drosophila* neuromuscular junction synapses

**DOI:** 10.1038/s41598-018-34250-9

**Published:** 2018-10-31

**Authors:** Kara R. Barber, Martin Hruska, Keegan M. Bush, Jade A. Martinez, Hong Fei, Irwin B. Levitan, Matthew B. Dalva, Yogesh P. Wairkar

**Affiliations:** 1 Neuroscience Graduate Program, University of Texas Medical Branch, Galveston, TX 77555 USA; 2 Department of Neurology & Mitchell Center for neurodegenerative diseases, University of Texas Medical Branch, Galveston, TX 77555 USA; 3 Department of Neuroscience, Vickie and Jack Farber Institute for Neuroscience, Thomas Jefferson University, Philadelphia, PA 19107 USA; 4 Department of Neuroscience, Cell Biology and Anatomy, University of Texas Medical Branch, Galveston, TX 77555 USA

## Abstract

Functional synaptic networks are compromised in many neurodevelopmental and neurodegenerative diseases. While the mechanisms of axonal transport and localization of synaptic vesicles and mitochondria are relatively well studied, little is known about the mechanisms that regulate the localization of proteins that localize to active zones. Recent finding suggests that mechanisms involved in transporting proteins destined to active zones are distinct from those that transport synaptic vesicles or mitochondria. Here we report that localization of BRP-an essential active zone scaffolding protein in *Drosophila*, depends on the precise balance of neuronal Par-1 kinase. Disruption of Par-1 levels leads to excess accumulation of BRP in axons at the expense of BRP at active zones. Temporal analyses demonstrate that accumulation of BRP within axons precedes the loss of synaptic function and its depletion from the active zones. Mechanistically, we find that Par-1 co-localizes with BRP and is present in the same molecular complex, raising the possibility of a novel mechanism for selective localization of BRP-like active zone scaffolding proteins. Taken together, these data suggest an intriguing possibility that mislocalization of active zone proteins like BRP might be one of the earliest signs of synapse perturbation and perhaps, synaptic networks that precede many neurological disorders.

## Introduction

Assembly of active zones on the presynaptic side of a synapse is one of the earliest steps in the formation of nascent synaptic communication networks^1,2^. Initiation of presynaptic assembly is accomplished, in part, by the transport of synaptic vesicle precursors (SVPs) and Piccolo-Bassoon transport vesicles (PTVs) that carry the components of active zones^2,3^. Interestingly, active zone density is maintained after its formation and decreases only later with aging^4^. Indeed, many synapses are thought to be stable for long periods of time after they are established^5^. Therefore, mechanisms must exist that maintain the presynaptic components such as active zones and postsynaptic density for their long-term stability. One way presynaptic components are maintained at the synapse is by active replenishment of synaptic proteins via axonal transport^6^. Although such mechanisms are relatively well studied for synaptic vesicles and mitochondria^7–9^, little is known about how cargo destined for active zones is transported and how the transport is regulated.

Par-1 kinase is a *Drosophila* homolog of Microtubule affinity regulating kinase (MARK), which is elevated in many diseases^10–12^ including, neurodevelopmental and neurodegenerative disorders^10,13–15^. At the synapse, Par-1, a cell polarity kinase^16^, has been previously implicated in regulating the postsynaptic glutamate receptor localization^17^. Interestingly, it was also noted that Par-1 is present in the presynaptic compartment albeit at very low levels^17^, suggesting that Par-1 may also have a role in the presynaptic compartment. It was recently demonstrated that elevated levels of presynaptic Par-1 lead to selective localization defects of BRP, with a significant accumulation of BRP within the axons and a corresponding decrease of BRP from the active zones^18^. While it is clear that the effect of increased Par-1 on localization of BRP is independent of Tau-a microtubule associated protein (MAP) and a well studied substrate of Par-1^18–21^, it is unclear whether other microtubule binding proteins such as Futsch (a MAP1B homolog)^22^, which has been proposed to be a likely substrate of Par-1^16^, might be involved. Also, it is unclear whether increased localization of BRP to the axons is a cause of the decreased BRP at the active zones. This is important because while the disruption of axonal transport has been implicated in many neurodegenerative diseases, it has been difficult to tease out whether axonal transport is a cause or consequence of synaptic demise^6^. In this report, using temporal expression of Par-1, we show that BRP accumulation precedes decreased BRP at the synapse and that it is independent on Futsch-the neuron specific MAP^22^. Interestingly, we find that increased levels of BRP in axons are accompanied by decrease in synapse function followed by an increase in “floating” T-bars- a electron dense structure present at active zones of invertebrates as well as vertebrates^23,24^, suggesting that active zones of these flies may be unstable. Finally, we show that BRP and Par-1 are present in the same complex raising the interesting possibility that presynaptic Par-1 may regulate the localization of BRP by interacting with it.

## Results

### Levels of Presynaptic Par-1 are important in determining the proper localization of BRP

**Figure 1:**
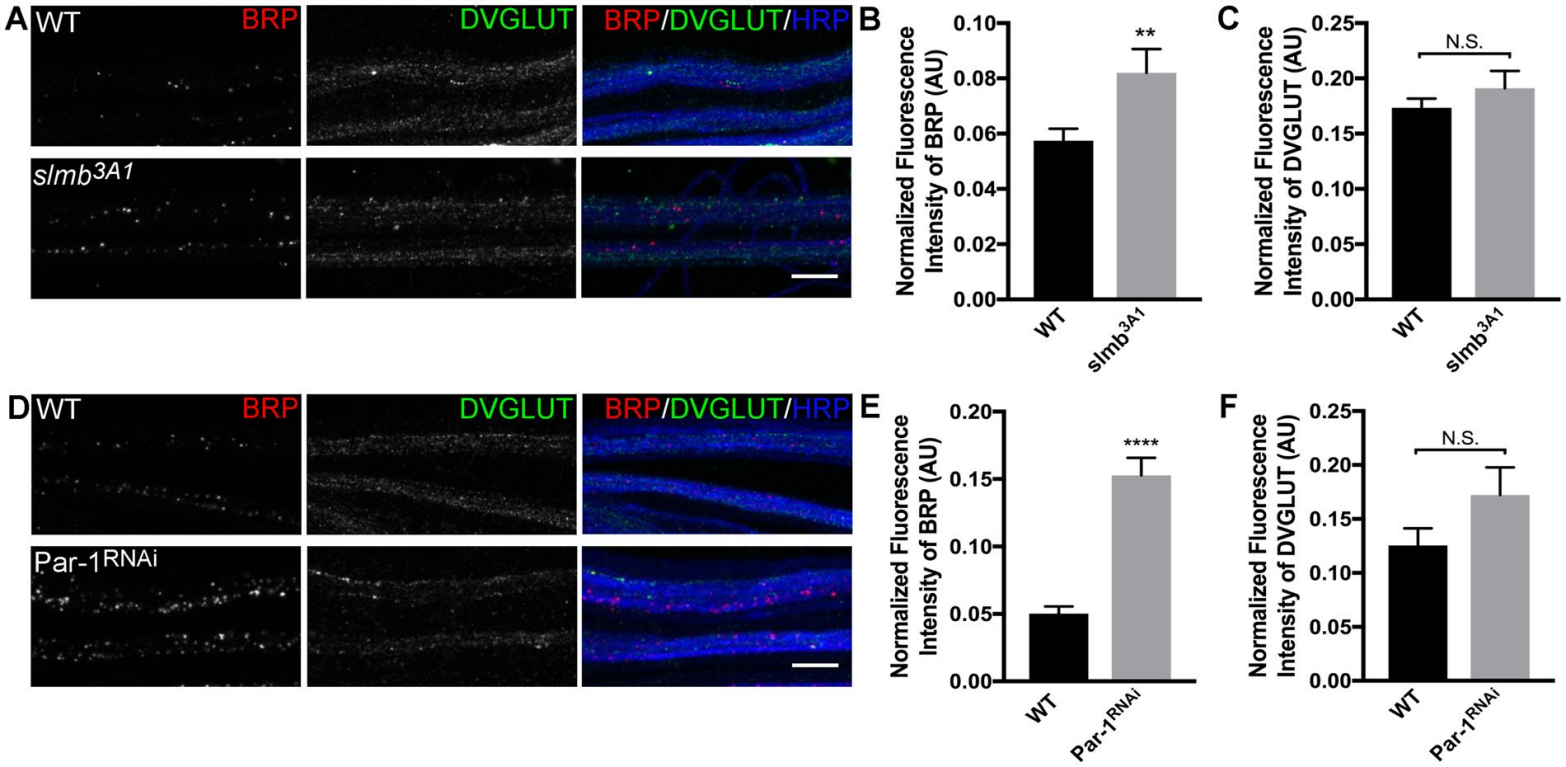
Precise levels of Par-1 are required for BRP localization. (**A**) Representative confocal stacks showing axon bundles from third instar larvae of WT and *Slmb* mutant (*slmb*^*3A1*^). Axon bundles are stained with antibodies against BRP (Red), DVGLUT (Green) and HRP (Blue), n > 10, Scale bar = 10 μm. (**B**) Mean fluorescent intensity of BRP (BRP fluorescence normalized to HRP) in axon bundles represented in A. (**C**) Mean fluorescence intensity of DVGLUT normalized to HRP intensity (**D**) Representative confocal stacks showing axon bundles from third instar larvae of WT and Par-1^RNAi^. Axon bundles are stained with antibodies against BRP (Red), DVGLUT (Green) and HRP (Blue), n > 10, Scale bar = 10 μm. (**E**) Mean fluorescence intensity of BRP normalized to HRP intensity. (**F**) Mean fluorescent intensity of DVGLUT normalized to HRP intensity. Error bars represent S.E.M. N.S. = p > 0.05, ^**^p ≤ 0.01, ^****^p ≤ 0.0001.

A previous study^18^ revealed that elevated levels of presynaptic Par-1 lead to a selective accumulation of BRP in the axons concomitant with loss of BRP from the synapses. Since this study largely used overexpression of Par-1 as a means to increase its levels, we wondered whether physiological manipulations that lead to increased Par-1 levels would also show selective axonal accumulations of BRP. To test this, we used well-characterized mutations in E3 ubiquitin ligase, Slimb (Slmb), which is known to increase the levels of Par-1^25^. Consistent with our hypothesis, mutations in *Slmb* led to a selective increase in the levels of BRP within the axons (Fig. 1A–C). Thus, the overexpression model of Par-1 has the same effect as physiologically increasing the levels of Par-1 by mutations in *Slmb*. Although it is important to note that the accumulation of BRP in *Slmb* mutants could be due to other possible downstream affects, the combination of increase in Par-1 levels in *Slmb* mutants^25^, and the selective increase in BRP suggests the possibility that increased Par-1 levels in *Slmb* mutants cause increased BRP accumulation within the axons.

Since increased levels of Par-1 caused increase in BRP accumulation in axons, we hypothesized that decrease in the levels of Par-1 would lead to a decrease in BRP levels in axons. To test this hypothesis, we knocked down Par-1 presynaptically using a previously characterized Par-1 RNAi line^17^ using the presynaptic driver BG380-Gal4^26^. Surprisingly, decrease in the presynaptic Par-1 also led to an increase in selective accumulation of BRP (Fig. 1D–F). RNAi knockdown of Par-1 with multiple presynaptic drivers yielded the same results (Supplemental Fig. 1A,B). These data indicate that not only does Par-1 have a physiological role in regulating the localization of BRP but also that its fine balance is required for the its precise localization.

### Accumulation of BRP precedes its decrease from synapses

**Figure 2:**
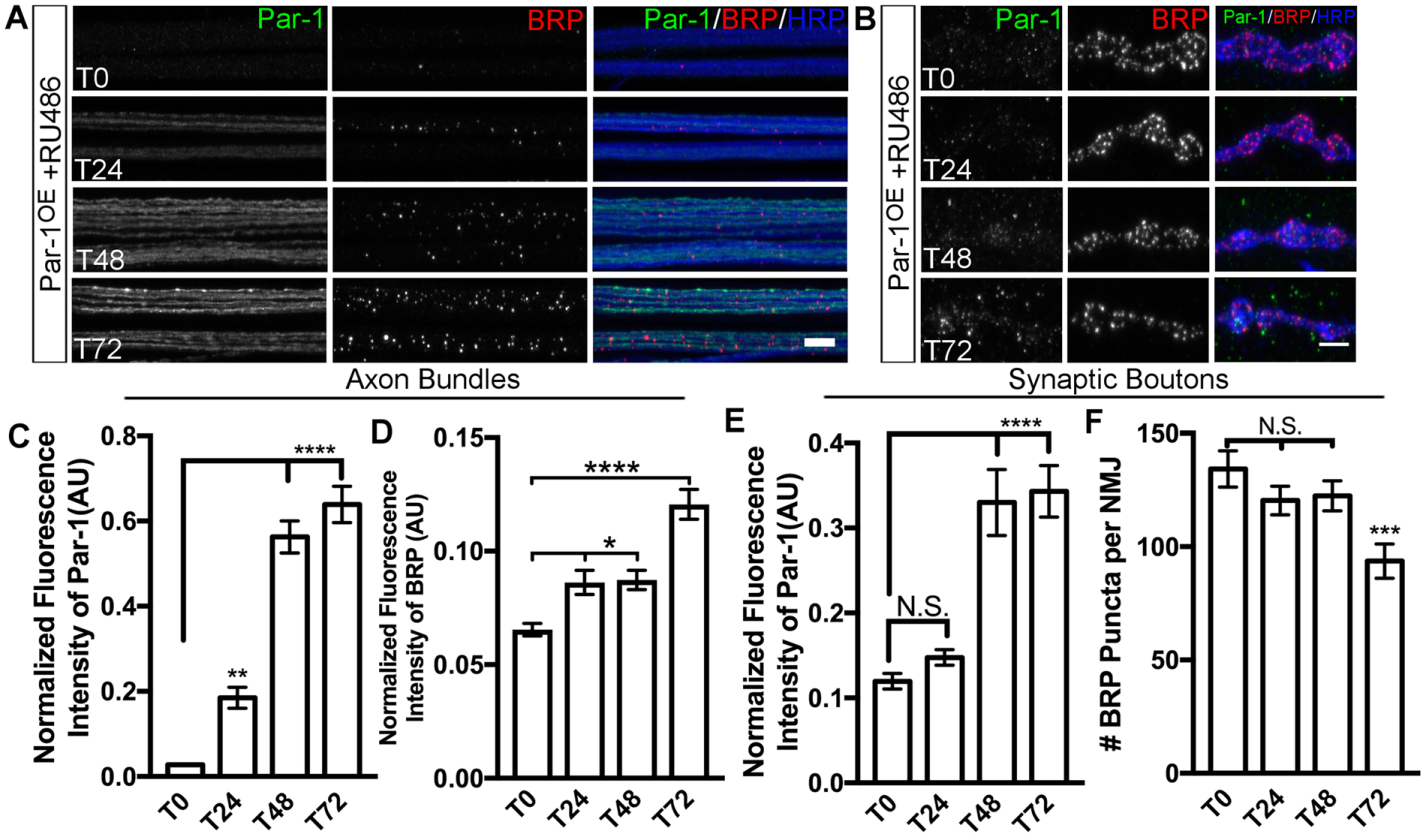
Accumulation of BRP in axons precedes its loss from synapses. (**A**,**B**) Representative axon bundles from larvae overexpressing Par-1 using GeneSwitch-Elav-Gal4. Time (T) represents time after exposure to RU-486 (T_0_, T_24_, T_48_, and T_72_) containing food. Axon bundles (**A**) are stained with antibodies against Par-1 (Green), BRP (Red) and HRP (Blue). Scale bar = 10 μm. Synaptic boutons (**B**) are stained with identical antibodies as A. Scale bar = 5 μm. (**C**) Mean Par-1 intensity normalized to HRP intensity within axons bundles (**D**) and mean BRP intensity normalized to HRP intensity in axon bundles (**D**) from same series of time points as A. n > 20. (**E**) Mean Par-1 intensity from synaptic boutons normalized to HRP (**F**) and quantification of the number of active zones (BRP puncta) per NMJ from same time points as B. n > 15. Error bars represent S.E.M. N.S. = p > 0.05, ^*^p ≤ 0.05, ^**^p ≤ 0.01, ^***^p ≤ 0.001, ^****^p ≤ 0.0001.

There are at least two possible reasons why BRP might accumulate within the axons. First, levels of neuronal Par-1 may determine proper transport BRP to the active zones. Second, precise levels of Par-1 may be required at the synapses to stabilize BRP at the active zones and breakdown of either of these processes may lead to BRP accumulating in the axons. If transport of BRP were the primary issue, we expect to find accumulation of BRP in axons to precede its reduction at the synapses. To test these possibilities, we took advantage of the GeneSwitch-Gal4 system^27^. This system allows the temporal expression of a transgene by feeding the larvae with a progesterone homolog, RU-486. In these experiments, we expressed the Par-1 transgene for a given period of time and then tested the effect of its expression on BRP accumulation within the axons and its loss from the synapses. Flies were allowed to lay eggs and develop on normal food and were transferred to food containing RU-486 at or just before the early third instar stage. Transfer onto the RU-486 containing food should turn “ON” the expression of Par-1 transgene. We systematically stained the larvae with antibodies against Par-1, BRP and HRP after 0, 9, 12, 24, 48 and 72 hours exposure to RU-486 and tested the expression of Par-1 transgene and the localization of BRP. Little to no detectable Par-1 was observed within axons from 0–9 hours (Supplemental Fig. 2A,B). The BRP intensity within the axons remained similar to the zero time point after the exposure to RU-486 (Supplemental Fig. 2A,C). We started to detect a significant increase in Par-1 within the axons at 12 hours (Supplemental Fig. 2A,B), along with a small but significant increase in the levels of BRP within the axons (Supplemental Fig. 2A,C). At 48 hours, the levels of Par-1 within the axons increased further (Fig. 2A,C) along with a significant increase in Par-1 levels at the NMJs (Fig. 2B,E). However, the number of BRP per NMJ area at 48 hours was unaltered (Fig. 2B,F); indicating that accumulation of BRP within axons precedes the detectable reductions of BRP from the synapses. Consistent with this idea, at 72 hours after induction of Par-1 we observed a significant reduction in the average number of BRP puncta at the NMJs (Fig. 2B,F). We also measured average synaptic span, and bouton numbers normalized to muscle area in the same larvae (Supplemental Fig. 3B,C) and found no change in these parameters to the control group. The intensity of BRP within the axons and at the number of BRP at the synapses were unaltered in the group that were exposed to RU-486 for the same time period as the experimental group but did not contain Par-1 transgene (Supplemental Fig. 4A–D). These data indicate that there was no “leaky” expression of Par-1 in the experimental group. Together, these data indicate that BRP accumulation precedes the loss of BRP at the synapses and thus points to the possibility that Par-1 may primarily regulate the transport of BRP.

### Synaptic function is altered before the loss of BRP from active zones

**Figure 3:**
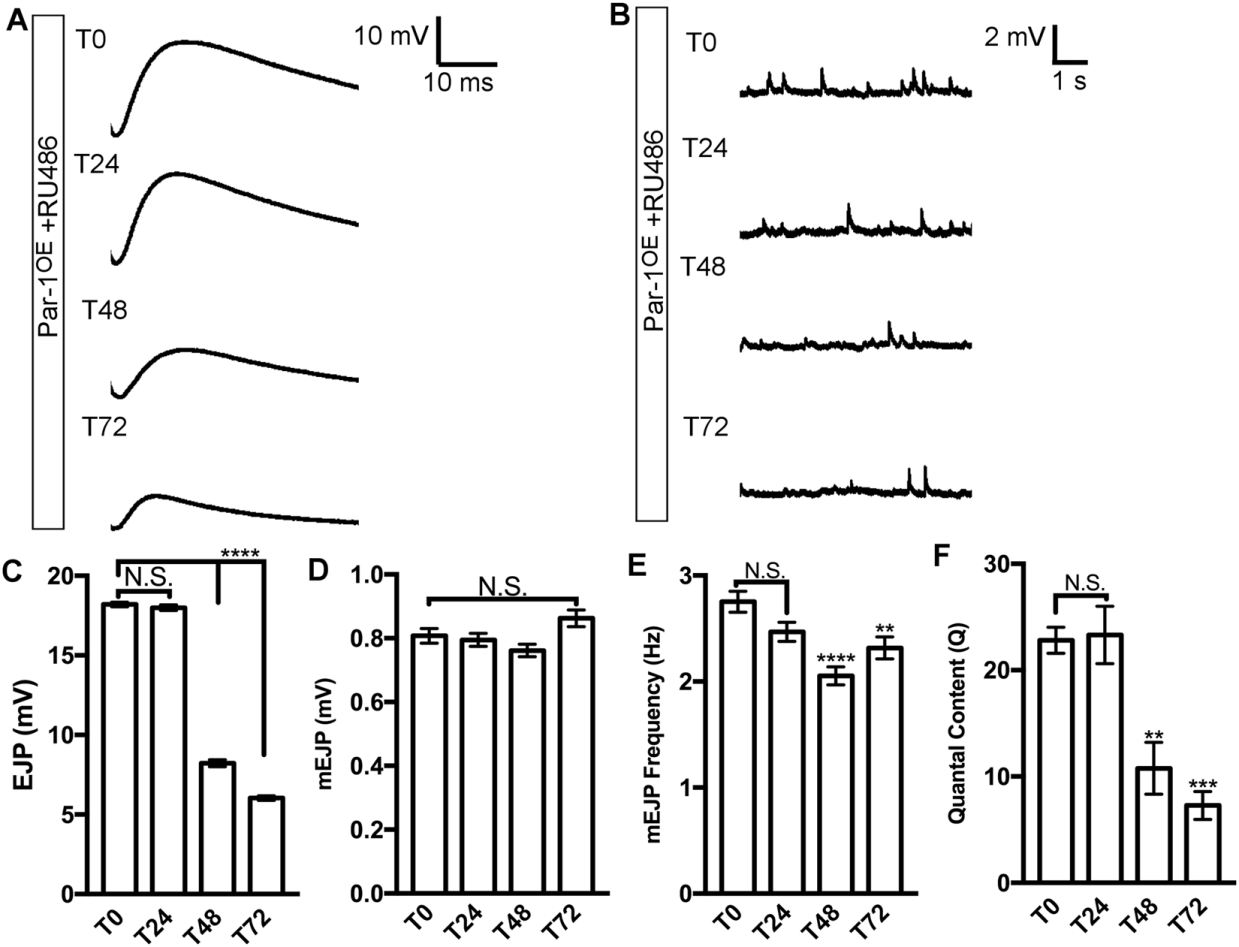
Functional deficits precede detectable decrease in BRP at synapses of flies overexpressing Par-1. (**A**,**B**) Representative traces of EJPs (**A**) and mEJPs (**B**) from larvae overexpressing Par-1 using GeneSwitch-Elav-Gal4. Time (T) represents time after exposing larvae to the RU-486 (T_0_, T_24_, T_48_, and T_72_). (**C**–**F**) Quantification of EJPs (**C**) and mEJP amplitudes (**D**), frequency (**E**), and quantal content (**F**) N = 8. Error bars represent S.E.M. N.S. = p > 0.05, ^**^p ≤ 0.01, ^***^p ≤ 0.001, ^****^p ≤ 0.0001.

To test whether decreased synaptic transmission^18^ was an early consequence of increased accumulation of BRP within axons, we performed intracellular recordings from larvae that had just begun to accumulate BRP (~24 Hours post induction of Par-1 transgene) and time points in between until the synapses started to show a significant decrease in BRP (~72 Hours). We found that at 0 hours when accumulation of BRP within the axons is not increased significantly, the synaptic transmission (mEJP amplitude, frequency and EJP amplitudes) (Fig. 3A–F) is indistinguishable from the controls (WT larvae raised on RU-486) (Supplemental Fig. 5A–F). Similarly, at 24 hours after the induction of Par-1 transgene although there was a significant increase in BRP levels within the axons, there was no change in the synaptic transmission. However, at 48 hours after the induction of Par-1 transgene, we began to observe a significant decrease in the EJP amplitudes and mini frequency while the mEJP amplitudes were unchanged (Fig. 3A–E). Interestingly, of note, at this time point there is a significant increase in the levels of Par-1 at the NMJs (Fig. 2B,E). However, the number of BRP puncta at the synapses were unaltered at this time point (Fig. 2B,F). At 72 hours after the induction of Par-1, there was a further decrease in EJP amplitudes while the mini EJP amplitudes remained unaltered (Fig. 3A–D), consistent with the previous observation that neither apposition nor the intensity of DGluRIII were significantly altered in lines overexpressing Par-1^18^. It is important to note while there were some effects of the drug RU-486 on mEJP amplitudes and frequency (Supplemental Fig. 4B,D,E), the EJP amplitudes and the quantal content remained unaltered (Supplemental Fig. 5C,F). These data show that disruption of synaptic transmission is an early consequence of increased BRP accumulation in axons.

### Axonal accumulation of BRP causes active zones to be unstable

**Figure 4:**
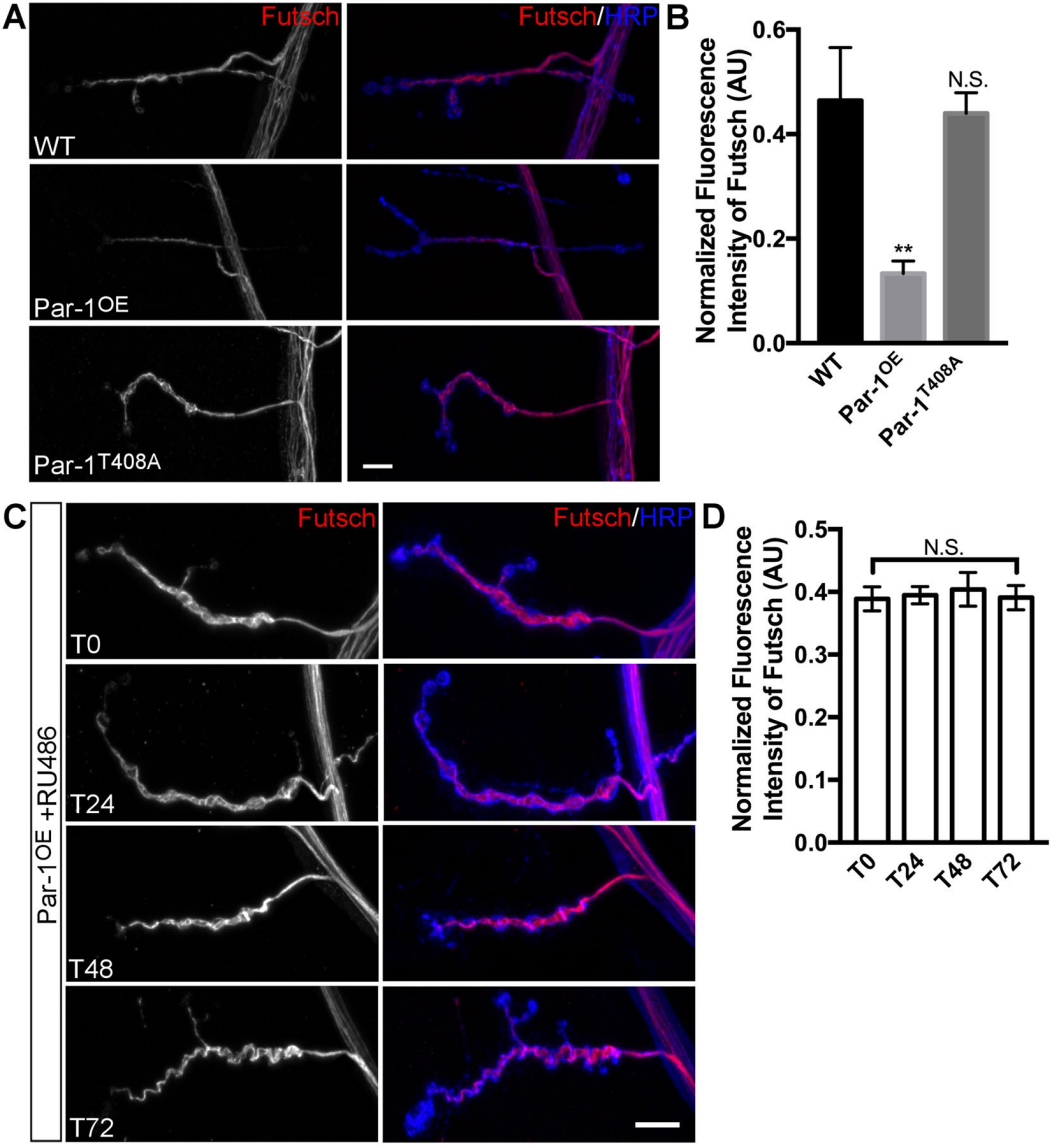
Futsch does not mediate accumulation of BRP within axons. (**A**) Representative confocal image stacks showing NMJ synapses from WT, Par-1^OE^ and Par-1^T408A^ third instar larvae stained with anti-Futsch (Red) and anti-HRP (Blue) antibodies. (**B**) Mean Futsch fluorescence intensity (A.U.) normalized to HRP intensity from entire NMJ arbor. n > 10. (**C**) Representative confocal stacks from larvae overexpressing Par-1 using GeneSwitch-ElavGal4 at T_0_, T_24_, T_48_, and T_72_. NMJ synapses are stained with anti-Futsch (Red) and anti-HRP (Blue) antibodies. (**D**) Mean Futsch fluorescent intensity (A.U.) normalized to HRP intensity from entire NMJ arbor. n > 20. Error bars represent S.E.M. N.S. = p > 0.05, ^**^p ≤ 0.01.

So far, our data indicate that decreased BRP at the synapses might be a consequence of axonal accumulation of BRP. If WT Par-1 levels are required for the proper localization of BRP to the active zones, increase in BRP within axons could cause active zones to be unstable by “starving” the active zones of “fresh” BRP. This could possibly compromise active zone integrity and make them unstable. Instability of synapses in *Drosophila* is often associated with a loss of microtubule binding protein Futsch^28^. Interestingly, a previous report has found that loss of Futsch leads to decrease in BRP density at the synapses and that Futsch interacts with BRP *in situ* at synapses^29^. Finally, Futsch has KXGS motif that can potentially be phosphorylated by Par-1 kinase^16^. Therefore, changes in the levels of Par-1 could alter the levels and/or localization of Futsch. To test these possibilities we stained the NMJ preparations from WT and Par-1 overexpressing flies with anti-Futsch antibodies. We observed no change in the intensity of Futsch within axons of flies overexpressing WT Par-1 (Supplemental Fig. 6A,B). Interestingly, however, there was a significant reduction in the intensity of synaptic Futsch (Fig. 4A,B). Importantly, such reductions were not apparent in Par-1^T408A^ expressing flies, indicating that the defect was not a result of secondary affect of Par-1 overexpression (Fig. 4A,B). To test whether the loss of Futsch might mediate affects of Par-1 overexpression, we tested whether *futsch* mutants accumulated BRP within their axons. Consistent with the previous report^29^, we did not observe axonal accumulation of BRP within the axons of *futsch* mutants (Supplemental Fig. 6C,D), indicating that Futsch may not mediate the affects of Par-1 overexpression. Finally, in the Gene Switch experiments (even at ~72 hrs post-induction of Par-1 transgene), we did not observe any alterations in the levels of synaptic Futsch (Fig. 4C,D) while there was a significant reductions of synaptic BRP (Fig. 2B,F). Although we cannot rule out the role of Futsch and/or cytoskeleton at later stages, these data indicate that Futsch, similar to tau^18^ is not required for the increase in BRP accumulation within axons at the initial time points.

**Figure 5:**
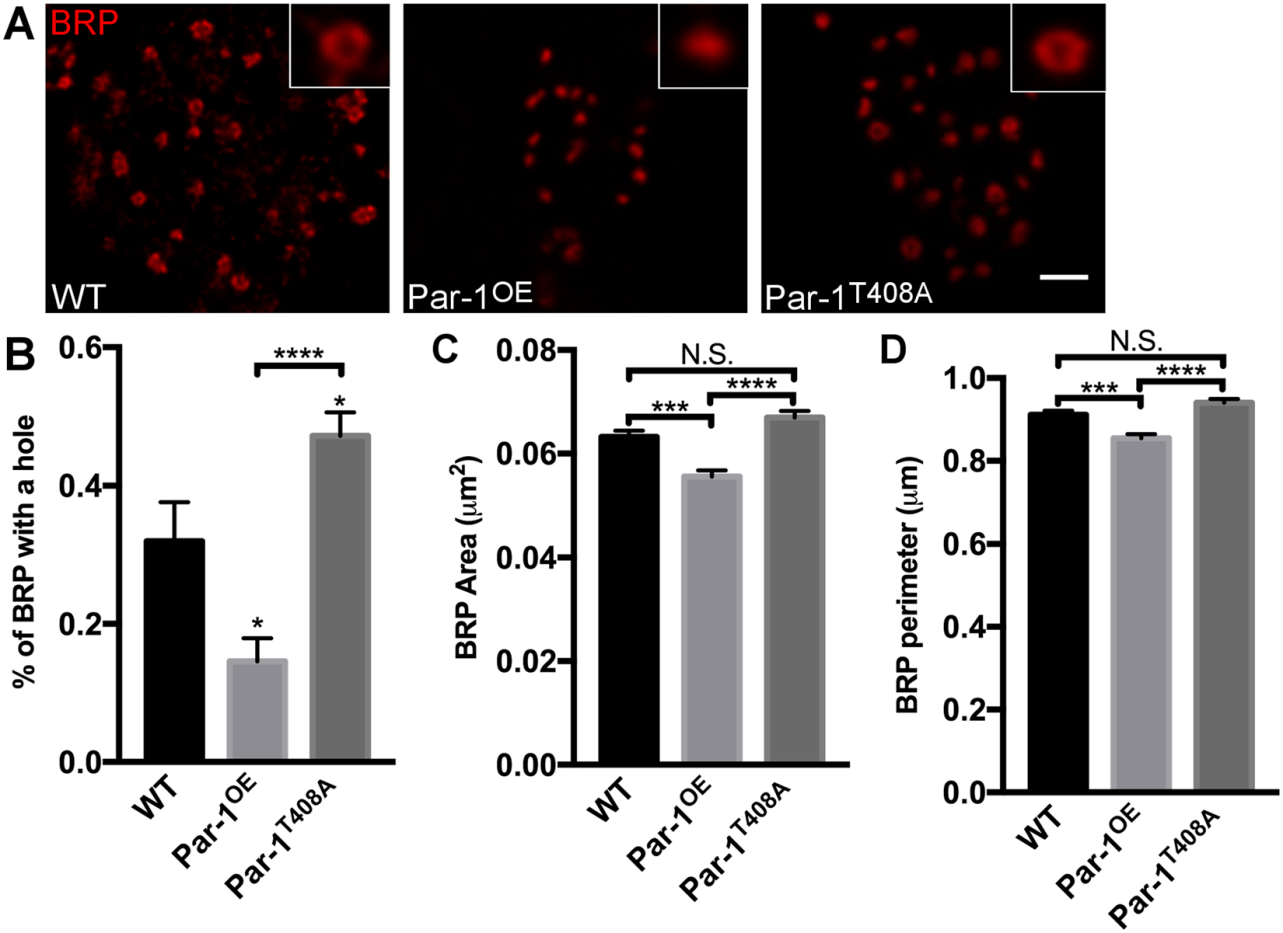
Elevated levels of Par-1 lead to alterations in BRP doughnuts. (**A**) Representative STED images showing BRP doughnuts at synapses from WT, Par-1^OE^ and Par-1^T408A^ third instar larvae stained against BRP. Scale bar = 1 μm. Insets highlight a representative BRP structure at boutons of the respective genotype. (**B**–**D**) Quantification of the percent BRP with doughnut structure (**B**), area of BRP puncta (**C**), and perimeter of BRP puncta (**D**). n > 700 BRP puncta count. Error bars represent S.E.M. N.S. = p > 0.05, ^*^p ≤ 0.05, ^***^p ≤ 0.001, ^****^p ≤ 0.0001.

Next, we reasoned that perhaps, the first signs of changes in active zone structure might manifest as subtle changes in the structure of T-bars. To test this possibility, we utilized simulated emission depletion microscopy (STED). When viewed using STED microscopy, BRP generally appears as a “doughnut” shaped structure at the active zones^30^. Subtle changes to this structure have previously been reported and are thought to be one of the early signs of active zone disassembly in a fly model of ALS^31^, perhaps by causing active zone instability. To test whether elevated levels of Par-1 lead to structural disruption of BRP doughnut structure, we performed STED on third instar larvae from WT, Par-1^OE^ and Par-1^T408A^ synapses. As expected, most BRP at the WT synapses showed the typical “doughnut” like structure, which was indistinguishable from larvae expressing Par-1^T408A^. However, Par-1 overexpressing active zones showed significant reductions in visible BRP doughnuts (Fig. 5A–D). These data indicate that synapses in Par-1 overexpressing flies might be unstable. It is interesting to note that although *futsch* mutants have decreased BRP density, the doughnut structure of BRP is indistinguishable from WT^29^ further supporting the idea that reductions in Futsch may not be the primary reason for the accumulation of BRP within axons.

**Figure 6:**
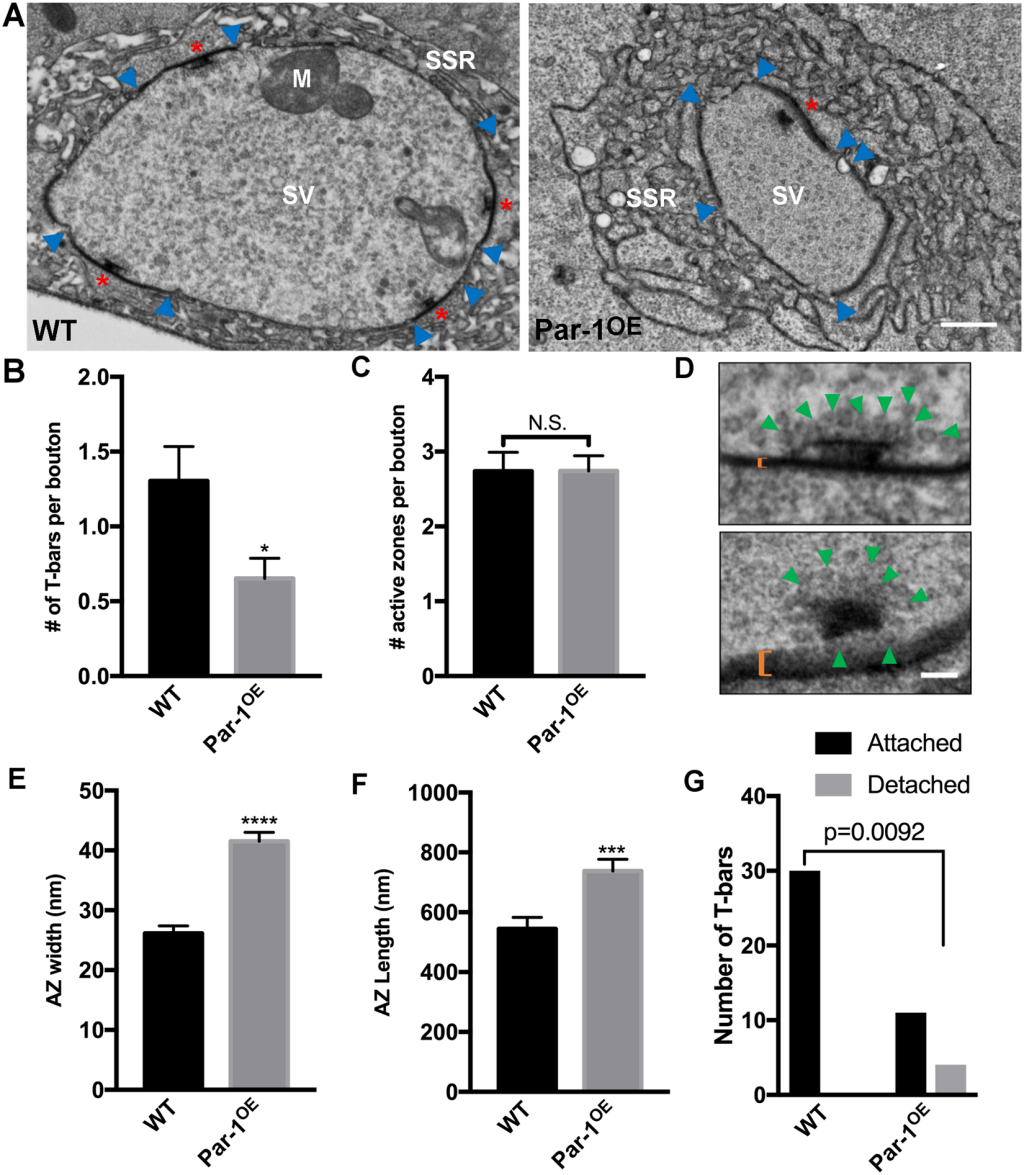
Elevated levels of Par-1 lead to disruption of active zone structure. (**A**) Representative electron micrographs from WT and Par-1^OE^ third instar larvae showing T-bars (red asterisks), synaptic vesicles (SV) and sub-synaptic reticulum (SSR). Scale Bar = 500 nm. (**B**) Mean number of T-bars per bouton (**C**) and AZ’s per boutons. (**D**) Representative T-bars with electron dense Active Zones (orange bracket), and synaptic vesicles (green arrows) from WT and Par-1^OE^. Scale bar = 100 nm. AZ width (**E**), and AZ length (**F**), from WT and Par-1^OE^ larvae. N = 20. (**G**) Quantification of detached T-bars in WT and Par-1^OE^. Par-1^OE^ larvae show a significant increase in detached T-bars. Error bars represent S.E.M. N.S. = p > 0.05, ^*^p ≤ 0.05, ^***^p ≤ 0.001, ^****^p ≤ 0.0001.

Since the STED data suggest that active zones in Par-1 expressing neurons might be unstable, we decided to test this directly by performing transmission electron microscopic analysis of active zones. An unstable active zone has previously been described at the ultrastructural level as diffuse and having long active zones with increased frequency of floating T-bars^28^. We found a significant increase in all these criteria in neurons expressing WT Par-1 (Fig. 6A–G). The length of active zones at Par-1^OE^ synapses as compared to WT was significantly increased. Furthermore, the electron-dense active zone regions in Par-1^OE^ flies were significantly more diffuse/wider than WT active zones, which were more tightly packed (Fig. 6D–F). While this pattern was consistent and was present in most sections from the EMs of the Par-1 overexpressing flies, it is possible that most of these sections are not from the middle of the bouton giving it a diffuse appearance. Finally, we observed there was a strong positive relationship between increased Par-1 and increase in the frequency of detached or floating T-bars (p = 009) (Fig. 5G). Together, these data indicate that synapses in Par-1 overexpressing flies are unstable.

### Par-1 associates with BRP in a complex

**Figure 7:**
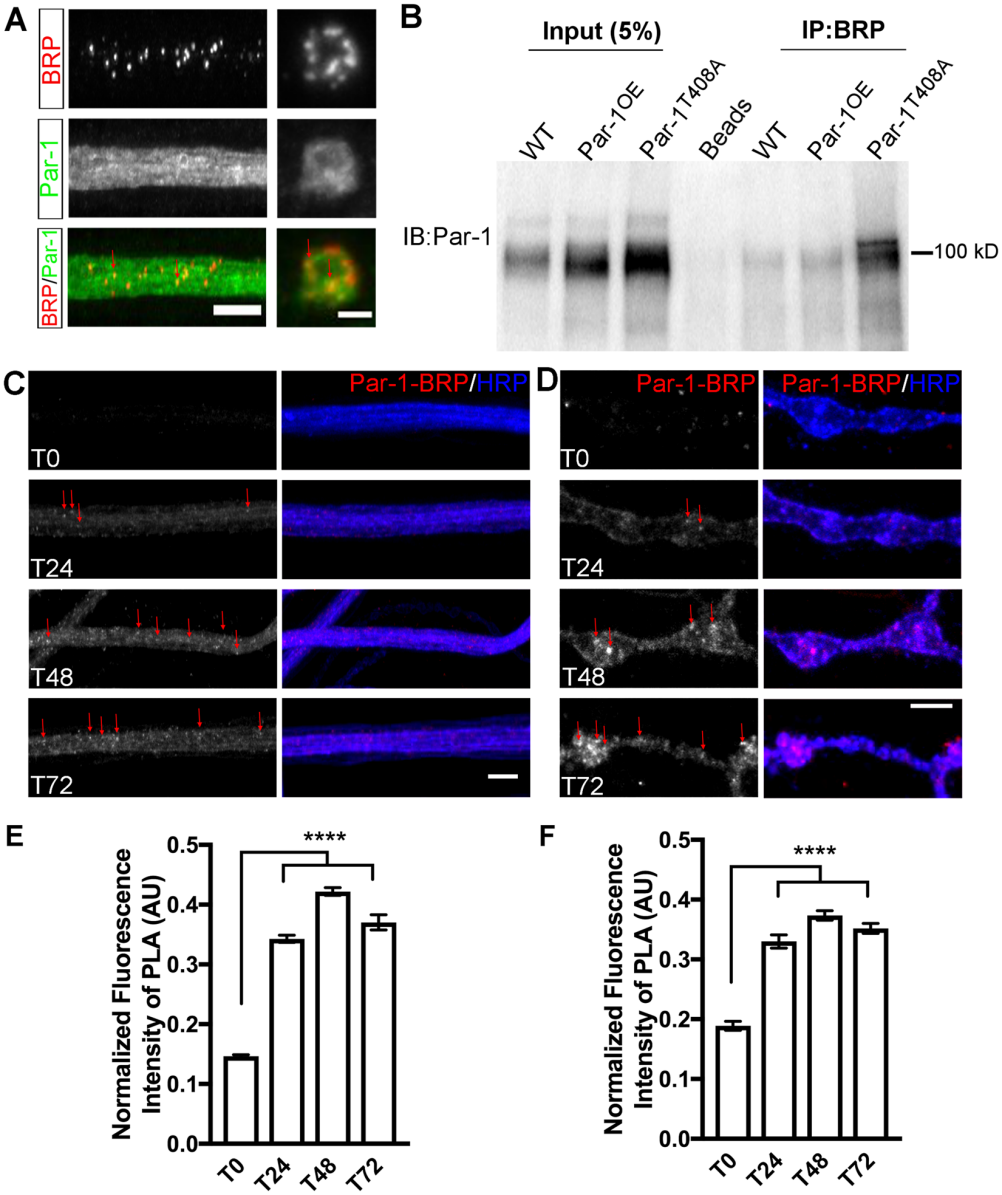
Par-1 and BRP are present within the same molecular complex. (**A**) Representative confocal stacks showing co-localization of Par-1 and BRP in the axons and boutons from third instar larvae of Par-1^OE^. Axons (Scale bar = 10 μm) and synaptic boutons (Scale bar = 5 μm) are stained with antibodies against BRP (Red), and Par-1 (Green). (**B**) Representative Western blot of proteins pulled down using the anti-BRP antibody and probed using anti-Par-1 antibody. Both the input and the IP were performed in the same blot and loaded on the same gel (different lanes). Beads only control shows no signal (**C**,**D**) Representative PLA signal (red) from larvae overexpressing Par-1 using GeneSwitch-ElavGal4 at T_0_, T_24_, T_48_, and T_72_ from axon bundles (**C**) and synaptic boutons (**D**). HRP marks presynaptic membrane boundary (blue) and arrows mark the punctate pattern and examples of brightest PLA signals. (**E**,**F**) Average PLA fluorescent intensity (A.U.) normalized to HRP intensity in axons bundles (**E**) and synaptic boutons (**F**). (n > 10). Error bars represent S.E.M. ^****^p ≤ 0.0001.

What might be the mechanism of action of Par-1? We have already explored two possible substrates of Par-1. Both these substrates of Par-1-Tau^18^ and Futsch (this study) do not seem to mediate the affect of Par-1 overexpression. Because BRP selectively accumulates in Par-1 expressing flies, we wondered whether one way Par-1 could selectively regulate BRP localization could be by interacting with it. To test this possibility, we first tested whether overexpressed Par-1 and BRP co-localized. We noted that overexpressed Par-1 and BRP were partially co-localized with each other in the axons and at the NMJs (Fig. 7A). However, co-localization is not a proof of interaction and one of the caveats of this experiment is that, co-localization can be attributed to the overexpression of Par-1, which saturates the axons and the synapses. Therefore, we went back to the geneswitch experiments where there was little to no detectable Par-1 at zero hours of Par-1 transgene induction (Fig. 2A–C,E), and performed the proximity ligation assay (PLA^29^,). PLA signal relies on proximity of two proteins to each other (<40 nm apart) such that the secondary antibodies that are conjugated to fluorescent oligonucleotides can be ligated giving rise to a bright fluorescent signal^32^. We used anti-BRP and anti-Par-1 antibodies to perform the PLA assays. At zero hours of Par-1 transgene induction we detected little to no PLA signal in axons or synapses consistent with the data that at zero hours we detect little to no of Par-1 expression (Fig. 7C,E). We detected a significant increase in PLA signal in axons at 24–72 hours (Figure C,E). This is consistent with the increase in Par-1 intensity in the axons for these time points (Fig. 2A,C). Interestingly, we also detected significant increases in the PLA signal at the synapse at 24 hours when the expression of Par-1 transgene was not detectably increased (Fig. 7D,F). This could be because PLA leads to a significant amplification of signal^33^. These data suggest that Par-1 is in a complex with BRP *in situ*. To further test this interaction under endogenous conditions, we performed co-immunoprecipitation assay with anti-BRP antibody. Protein extracts from the fly heads were used to test this interaction. BRP successfully immunoprecipitated Par-1 from WT heads indicating that Par-1 and BRP are present in the same protein complex (Fig. 7B and Supplemental Fig. 7). BRP also immunoprecipitated overexpressed Par-1 and surprisingly, overexpressed Par-1^T408A^. These data indicate that even inactive Par-1 can interact with BRP and show that Par-1 and BRP are in the same molecular complex (Fig. 7B). There was no signal in the beads only control (Fig. 7B). Together, these data indicate that Par-1 and BRP are in the same complex and that Par-1 and BRP may share a functional relationship.

## Discussion

Par-1 is an evolutionarily conserved serine threonine kinase that has many diverse roles, including important roles in regulating cell polarity and regulating microtubule stability^16,34^. Genome-wide association studies have implicated Par-1 (MARK) in Alzheimer’s disease (AD)^13^. While accumulations of Aβ and tau are implicated in the widespread neuronal death found in late stages of AD, synapse instability is often associated with early stages during the progression of AD^35,36^. Indeed, animal models of tauopathy show an increase in synapse instability^37^. Therefore, we propose that synapse instability might be one of the early events in neurodegenerative diseases like AD and that increase in Par-1/ MARK4 could facilitate the instability and hasten the demise of synapses.

Synaptic plasticity is determined by its ability to modulate its response to stimulation^38^. Generally, activity leads to strengthening of synapses, which is bigger response to stimulation^39^. Therefore, maintenance of synapses is important in maintaining the synaptic networks, which are disrupted in both neurodevelopmental and neurodegenerative diseases^40–42^. Indeed, mutations in cysteine string protein (CSP), which plays an important role in synaptic maintenance, causes a progressive motor neuron disorder characterized by neurodegeneration^43^. Thus, maintaining stable synapses might be important to avoid the failure of synaptic networks.

At the *Drosophila* NMJ synapses, active zones can be rapidly modified to induce synaptic homeostatic changes, which are partly dependent on BRP^44^. Interestingly, in a *Drosophila* model of ALS, disruption of shape and size of T-bars, which consists primarily of BRP, precedes synapse degeneration^31^. These data suggest that disruption of T-bars might be an early marker for synapse breakdown^31^. Our data support this hypothesis because we find that the doughnut shape of T-bars is dramatically altered in flies overexpressing Par-1 and this happens before the decrease in the number of AZs marked by BRP. Finally, we posit that loss of BRP from synapses could lead to a failure of synaptic homeostasis because BRP plays an important role in synaptic vesicle release^30^. Interestingly, loss of synaptic homeostasis has been implicated in early phases of neurodegeneration^45^ and, restoring synaptic homeostasis can restore synaptic strength in a *Drosophila* model of ALS^46^. Thus, gradual loss of BRP from synapse may impair the ability of a synapse to efficaciously respond to changes that perturb synaptic homeostasis leading to catastrophic failure of neural networks^40^.

### Role of Par-1 in regulating synapse maintenance

One of the vital functions performed by axonal transport is to maintain steady state levels of synaptic proteins required for the efficacious release of neurotransmitter release^47,48^. Disruption of axonal transport has been implicated in neurodegenerative diseases^6^. Indeed, mutations that affect axonal transport lead to neurodegenerative diseases^49^. A recent study suggests that active zone density is maintained during the developmental stages but is significantly decreased with aging^4^. Interestingly, axonal transport also declines with aging^50^ suggesting that a combination of decreased axonal transport of active zone proteins along with aging may lead to a gradual decrease in the maintenance of active zones. This may eventually lead to a failure to maintain synaptic function and ultimately lead to synapse degeneration. While this hypothesis is generally accepted, it has proven difficult to determine whether axonal transport is a cause or consequence of synapse loss. Our temporal analysis suggests that following sequence of events: Par-1 localizes to the axons followed by BRP accumulation in axons likely leading to the decreased synaptic function and finally the reduction of BRP from synaptic active zones likely leading to synapse instability. Together, these findings support the hypothesis that defects in axonal transport cause synapse degeneration.

### How does Par-1 regulate localization of BRP?

While so far we do not precisely understand how active zone scaffold proteins like BRP are localized, based on our present study, we can speculate that phosphorylation of Par-1 substrate may be important in determining the localization of BRP. This is because while the expression of WT Par-1 causes accumulation of BRP within axons, expression of inactive Par-1 does not lead to show any aberrant localization of BRP. Our data suggest that defects in BRP localization are not mediated either by tau^18^ or Futsch (this study) but BRP may be a possible substrate of Par-1. This is because our data indicate that BRP and Par-1 may be in the same molecular complex. However, it remains to be determined whether Par-1 can phosphorylate BRP and whether phosphorylation of BRP is required for its localization. Previous studies have shown that BRP can be acetylated, and that this posttranslational modification is important in regulating the structure of T-bars^51^ but whether BRP can be phosphorylated remains to be studied. Finally, our data indicate that presynaptic Par-1 levels are important in determining BRP localization because Par-1 knockdown also results in the accumulation of BRP within the axons. Thus, Par-1 not only has an important role in postsynaptic compartment^17^ but also has an important function on the presynaptic side. Finally, it should be noted that this study is a limited but an important extension of our previous study of how Par-1 regulates the localization of important active zone proteins such as BRP^1^. Our study also opens up a lot of questions. For example, what is the half life of BRP at the AZs? Does BRP get replaced? If so, at what rate? These are some important questions that should be addressed by future studies but our study opens up the possibility to study these processes in much more detail.

## Materials and Methods

### Fly Stocks

Flies were reared at 25 °C in medium containing Nutri-Fly^TM^ Bloomington formulation (Genesee Scientific, San Diego, CA), Jazz mix (Fisher Scientific, Waltham, MA, USA), sugar and powdered yeast (Genesee Scientific) in an 8:5:1:1 ratio and made according to standard procedures. The following fly stocks were used in this study: UAS-Par-1, UAS-Par-1^T408A^, and UAS-Par-1^RNAi^ (All gifts from Bingwei Lu, Stanford School of Medicine, Stanford, CA, USA^25^), *slmb*^3A1^ (Bloomington Stock Center)^52^, *olk*^1^ and *olk*^3^ (a gift from Doris Kretzschmar, Oregon Health and Science University, Portland, OR, USA)^53^. The following GAL4 lines were used: BG380-Gal4 (A gift from Aaron DiAntonio, Washington University Medical School, St. Louis, MO, USA)^26^, and ELAV-*GeneSwitch* (Bloomington Stock Center)^27^.

### RU486-GeneSwitch experiments

All experiments using the RU486-GeneSwitch system were performed according to Osterwalder *et al*.^27^. For overexpression of Par-1, UAS-Par-1 and ELAV-GeneSwitch adults were placed on normal food and allowed to mate for two days at 25 °C. Late second instar larvae or early third instar larvae were then placed on RU486 containing food (20 μg/ml RU486 diluted in EtOH)(Mifepristone; Sigma, St. Louis, MO) and dissected at time points following RU486 exposure, T_0_, T_24_, T_48_, and T_72_. Dissections, imaging, electrophysiology and analyses for these experiments are described in the following sections.

### Immunohistochemistry

Larvae were dissected and stained as previously described^54^. Briefly, the larvae were dissected in cold 1X PBS solution followed by fixation in Bouin’s fixative for 5 minutes. Larvae were washed 3X with PBS-Triton (0.1% solution) and blocked using 5% NGS solution in PBS. Following primary antibodies were used: anti-BRP (1:250, DSHB, Iowa city, IA)^55^, anti-Futsch (1:100, DSHB, Iowa City, IA)^22^, anti-DVGLUT (1:10,000, gift from Aaron DiAntonio, Washington University in St. Louis, MO)^56^ and anti-Par-1 (1:1,000, gift from Bingwei Lu, Stanford School of Medicine, CA)^17^. Dylight conjugated goat anti-HRP antibody (1:1,000), Goat Cy3-, and Alexa 488 conjugated secondary antibodies against mouse and rabbit IgG (1:1000) were obtained from Jackson ImmunoResearch, West Grove, PA.

### Confocal microscopy and analysis

Imaging and analysis of intensity of proteins within axons was performed as described previously^57^ using a Nikon C1 confocal microscope. To compare different genotypes, samples were processed simultaneously. Imaging was performed on the same day and same slide, with an appropriate control. Each staining was repeated at least three times with at least four larvae per genotype and at east 10 NMJs per individual experiment were included in the analyses.

For quantification of intensities within axon bundles and NMJs, a complete z-series stack collected at intervals of 0.4 μm was projected using the maximum intensity method. Staining intensities of various proteins within the axon bundles and the NMJs were quantified by using MetaMorph software (Molecular Devices, Sunnyvale, CA, USA). For axon bundles and synaptic boutons, HRP was used to set the color threshold. Only the axonal compartment and the region of synaptic bouton determined by HRP staining were used to measure the intensity of the red, green and blue channels. Intensity measurements at boutons were taken across the entire NMJ arbor. At axon bundles, intensity measurements were taken from axon bundles passing over the segments A3-A4^57,58^. Measurements were taken from a box of 50 μm^2^ and 3 random samples were taken per images, with a total of at least 10 images per genotype per experiment, which was repeated three times^57,58^. Intensity measurements at boutons and within axon bundles were normalized to HRP intensity. Quantification of active zones (BRP) at synaptic boutons and bouton size was performed from the entire NMJ arbor and the number of BRP was counted manually and the count was tracked using Fiji^59,60^. Bouton number and synaptic span were normalized to the mean muscle surface area of each genotype. Synaptic span was quantified using Simple Neurite Tracer plugin in Fiji^59^. Experimenter was blinded to the genotypes of the larvae while performing and analyzing the experiments.

### Electrophysiology

Intracellular electrophysiological recordings were performed on muscle 6, segment A3-A4 as previously described^61^. Dissections and intracellular recording were performed in HL3 saline^62^ containing 0.45 mM Ca2+. The cells with input resistance of at least 5 MΩ and resting membrane potentials of less than -63mV were used for analyses. Mean EJP amplitudes, mEJP amplitudes and frequency, were calculated from 75 consecutive traces or events using *pClamp* 9 *software* (Molecular Devices). Quantal content was estimated by dividing the mean EJP amplitude by the mean mEJP amplitude (EJP/ mEJP) from the same synapse. For GeneSwitch experiments recordings were performed within a 2-hour window around the time point indicated in figures. A total of 5 recordings from 5 larvae per genotype were made per experiment and the experiment was repeated three times.

### Electron Microscopy

Transmission electron microscopy (TEM) was performed on wandering third instar larvae as previously described in Barber *et al*.^18^. EM sections were obtained using a JEOL 1200EX microscope. Sections analyzed were all mid bouton sections from 1b boutons and showed clear SSR and synaptic vesicles. T-bars, AZ count, AZ length, and AZ width were quantified using Fiji distribution in ImageJ^59^. Quantification for each genotype was performed on N of 20 or more synaptic boutons from at least 4 larvae per genotype. Floating T-bars were counted manually, and the experimenter was blinded to the genotype. Floating T-bars were defined as having at least a few synaptic vesicles localizing between the T-bar structure and the electron-dense AZ.

### Proximity Ligation Assay (PLA)

Third instar larvae were dissected in cold HL3 solution^62^ and were incubated with anti-BRP (1:250, DSHB, Iowa city, IA)^55^ and anti-Par-1 antibodies (1:10,000, gift from Bingwei Lu, Stanford School of Medicine, CA) overnight at 4 °C. Cy5-conjugated anti-HRP antibody raised in Goat was used (Jackson ImmunoResearch) at 1:500 to label the neuronal membranes. For PLA, Duolink Mouse Rabbit *in situ* PLA kit (Sigma-Aldrich, St. Louis, MO) was used and the PLA assay was performed as previously described^29,63^. Synaptic boutons and axon bundles passing over A3–4 were imaged using Nikon C1 confocal microscope and analyzed as described above. At least 4 larvae from each time point and 10 NMJs were analyzed. Analysis of average PLA signal intensity was performed using MetaMorph software (Molecular Devices, Sunnyvale, CA, USA) as described in the confocal microscopy analysis section (above) and normalized to HRP intensity.

### Stimulated emission depletion microscopy and analysis

Stimulated emission deletion microscopy (STED) on NMJ preparations and analysis of BRP structure was done as previously described in Shahidullah *et al*.^31^. Images were taken of type 1b boutons from muscle 4 segments A3–4. BRP doughnuts at synaptic boutons were defined as having a “doughnut shape” when a hole could be visualized in the center of BRP puncta and were counted manually and the count was tracked using Fiji^59^. Images analyzed were maximum projections. Perimeter and area of BRP puncta at synaptic boutons were quantified using particle analysis in Fiji^59^. At least 4 larvae and 10 NMJs were used in this analysis.

### Co-immunoprecipitation

Frozen (−80 °C) WT, Par-1^OE^ and Par-1^T408A^ adult flies were vortexed and the vortexed (Separated parts) while still frozen, were passed through a sieve (No. 40) to separate the heads. At least 100 heads were collected per genotype and used for the Co-IP experiment. Heads were homogenized mechanically in 200 μl of lysis buffer^64^ and incubated at 4 °C for one hour. The head lysate was then incubated with anti-BRP antibody (1:25) overnight at 4 °C. BRP along with its binding partners were isolated by incubating with Dynabeads (Invitrogen) for 1–3 hours at 4 °C. After washing and elution, the samples were resolved using 4–20% gradient SDS-PAGE gel followed by western blotting. Blots were then probed using anti-Par-1 antibody (1:8,000) followed by HRP-conjugated goat α-rabbit (Jackson ImmunoResearch) secondary antibodies (1:3000). The blot was immersed in BIORAD clarity western ECL blotting substrate and images were acquired using *Bio*-*Rad’s ChemiDoc* XRS + system.

### Statistical analysis

Experimenters were generally blinded to the genotypes. Analyses were performed on at least 4 larvae per genotype for a single experiment and each experiment was repeated at least 3 times. Statistical analyses and graphs were generated using GraphPad Prism (GraphPad Software, Inc.). Student’s T-test was used to compare within two groups or one-way ANOVA followed by Dunnett’s or Tukey’s post-hoc tests were performed to compare means between three or more groups. Fisher’s exact test was used to test the occurrence of detached T-bars in WT and Par-1^OE^. P values less than 0.05 were considered significant. However, in most cases the p values obtained in this study were less than 0.01.

## Electronic supplementary material

Supplementary Information
