# Supplementary material for: Levels of Par-1 kinase determine the localization of Bruchpilot at the Drosophila neuromuscular junction synapses
Source: Sci Rep. 2018 Oct 31;8:16099. doi: 10.1038/s41598-018-34250-9 (PMC6208417; doi:10.1038/s41598-018-34250-9)
Supplement: Supplementary file 1 — Supplementary Information [file 41598_2018_34250_MOESM1_ESM.pdf]

Manuscript Title:

Levels of Par-1 kinase determine the localization of Bruchpilot at the *Drosophila* neuromuscular junction synapses

Authors:

Kara R. Barber[1, 2], Martin Hruska[3], Keegan M. Bush[1,2], Jade A. Martinez[2], Hong Fei[3], Irwin B. Levitan[3], Matthew B. Dalva[3], and Yogesh P. Wairkar[1, 2, 4*]

1: Neuroscience Graduate Program, University of Texas Medical Branch, Galveston, TX 77555

2: Department of Neurology & Mitchell Center for neurodegenerative diseases, University of Texas Medical Branch, Galveston, TX 77555

3: Department of Neuroscience, Vickie and Jack Farber Institute for Neuroscience, Thomas Jefferson University, Philadelphia, PA 19107

4: Department of Neuroscience, Cell Biology and Anatomy, University of Texas Medical Branch, Galveston, TX 77555

*: Corresponding Author. Email: yowairka@utmb.edu

Supplemental Figures

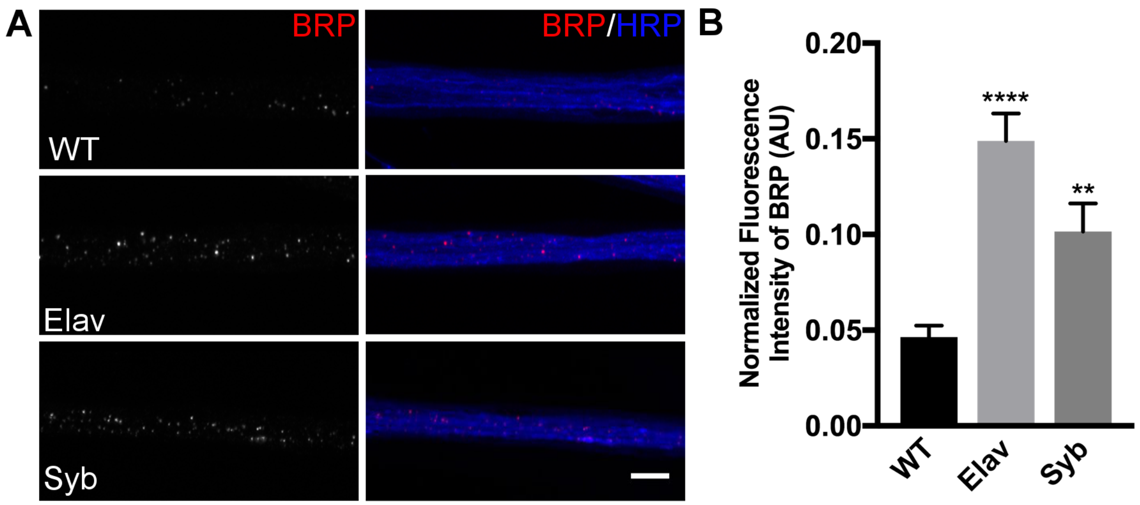

Supplemental Figure 1

Knockdown of Par-1 using multiple presynaptic drivers show accumulation of BRP in axons

(A) Representative confocal stacks showing axons from third instar larvae of WT and Par-1^{RNAi} third instar larvae using different presynaptic Gal-4 drivers (indicated on figure). Axons are stained with antibodies against BRP (Red), and HRP (Blue). Scale bar = 10μm **(B)** Mean fluorescence intensity of BRP normalized to HRP. Error bars represent S.E.M. **=p≤0.01, ****=p≤0.0001.

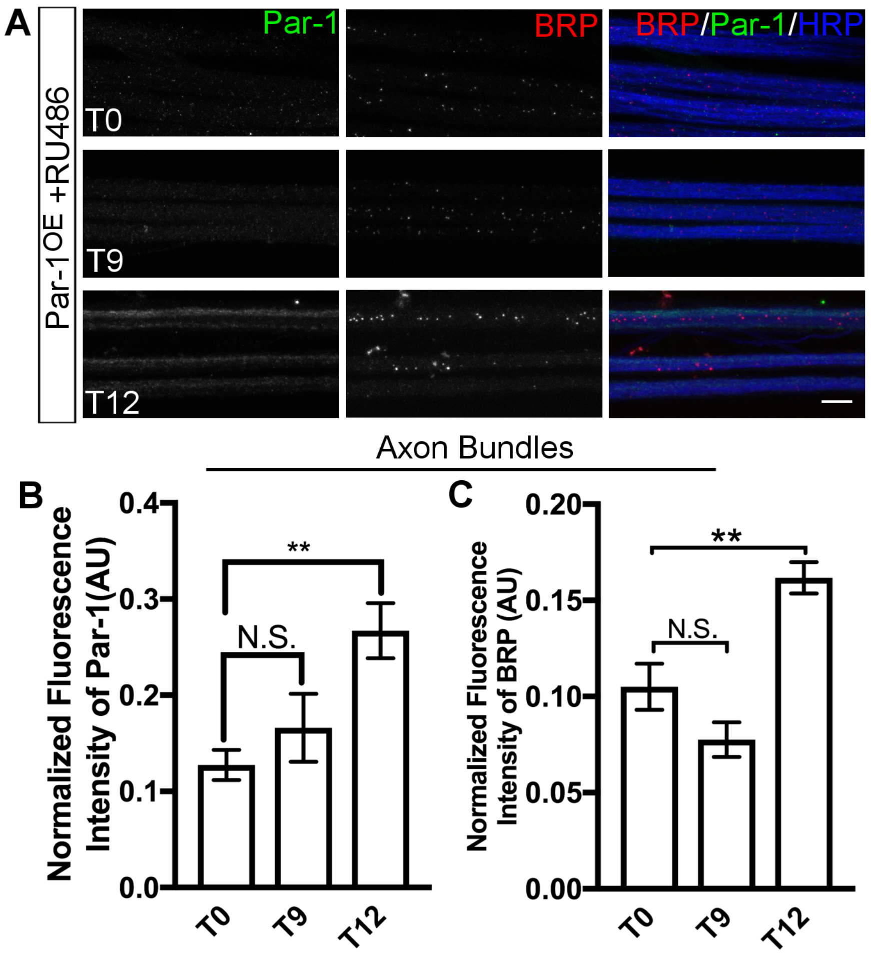

Supplemental Figure 2

No change is noticeable in the intensity of axonal BRP until 12 hours of Par-1 induction

(A) Representative confocal stacks from larvae overexpressing Par-1 induced using GeneSwitch-ElavGal4 from T_0, T_9, and T_{12}. Axons are stained with antibodies against Par-1 (Green), BRP (Red) and HRP (Blue). Mean fluorescence intensity of Par-1 (**B**) and BRP (**C**) normalized to HRP in axon bundles from T_0, T_9, and T_{12}. N=12. Error bars represent S.E.M. N.S.=$p>0.05$, **=$p\leq0.01$, ***= $p\leq0.001$, ****=$p\leq0.0001$.

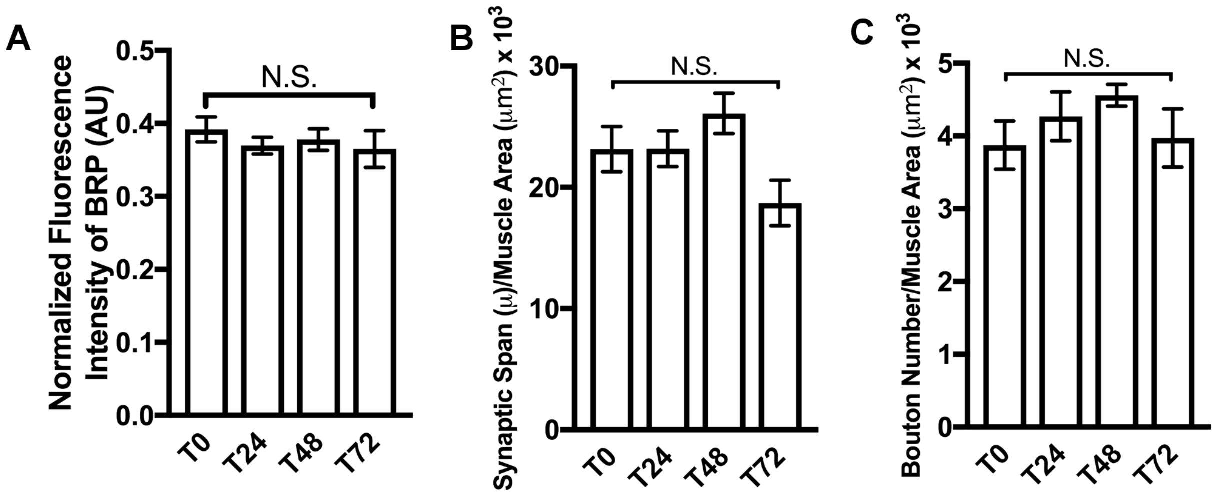

Supplemental Figure 3

Additional quantification pertaining to Figure 2

(A) Mean fluorescence intensity of BRP normalized to HRP from synaptic boutons, across the NMJ arbor from larvae overexpressing Par-1 using GeneSwitch-Elav-Gal4. Time (T) represents time after exposing late second instar larvae to the RU-486 (T_0, T_{24}, T_{48}, and T_{72}). **(B-C)** Quantification of synaptic span **(B)** and average number of boutons per NMJ **(C)** from identical genotypes and normalized to mean muscle area. N≥20, N.S.=$p>0.05$.

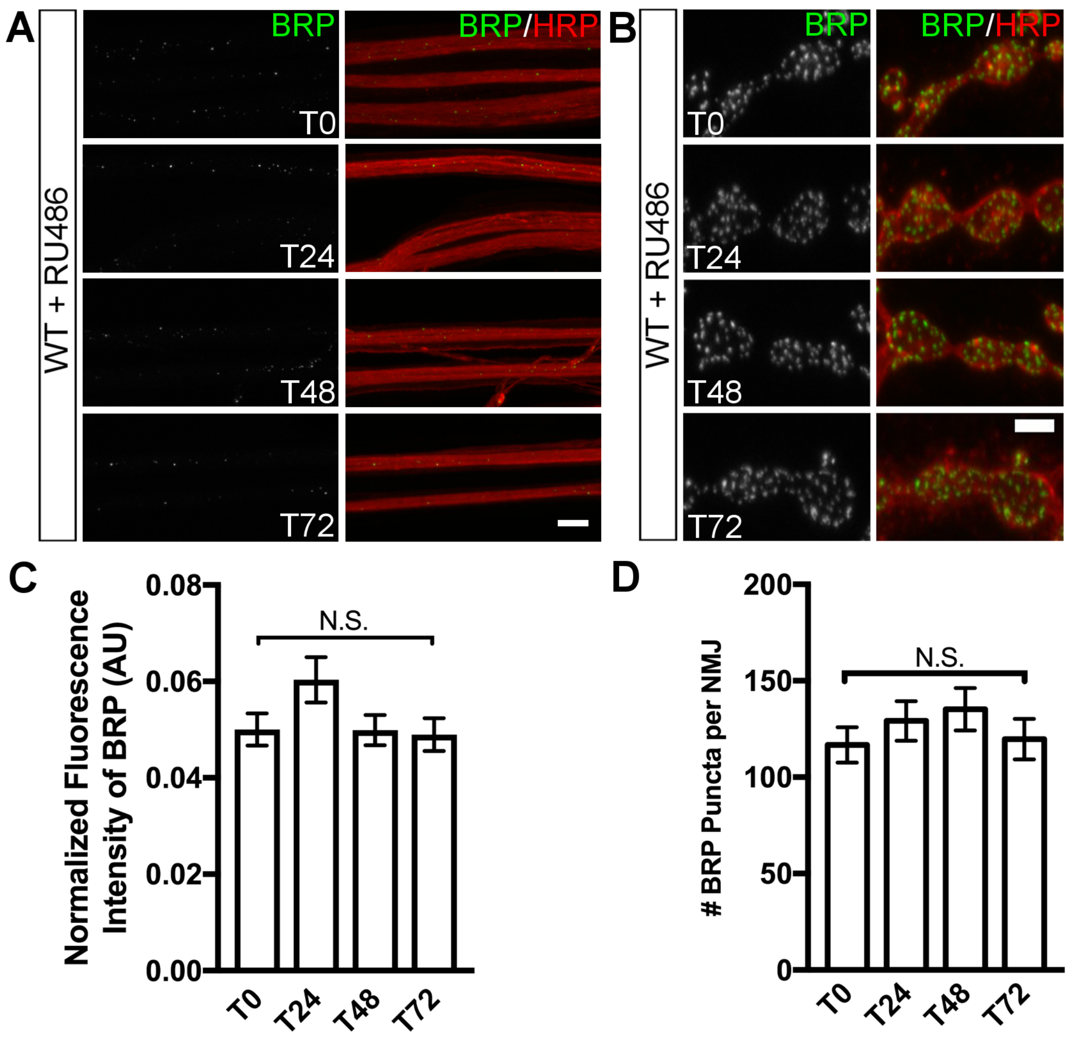

Supplemental Figure 4

RU486 alone does not lead to BRP accumulation or loss of BRP at synapses

(A-B) Representative confocal stacks from WT larvae. Time (T) represents time after exposing the early third instar larvae to the RU-486 containing food (T_0, T_{24}, T_{48}, and T_{72}). Axons **(A)** and synaptic boutons **(B)** are stained with antibodies against BRP (Green) and HRP (Red). **(C-D)** Mean fluorescence intensity of BRP normalized to HRP **(C)** and BRP puncta count per NMJ **(D)** from identical genotypes in A-B. N=12. Error bars represent S.E.M. N.S.=$p>0.05$.

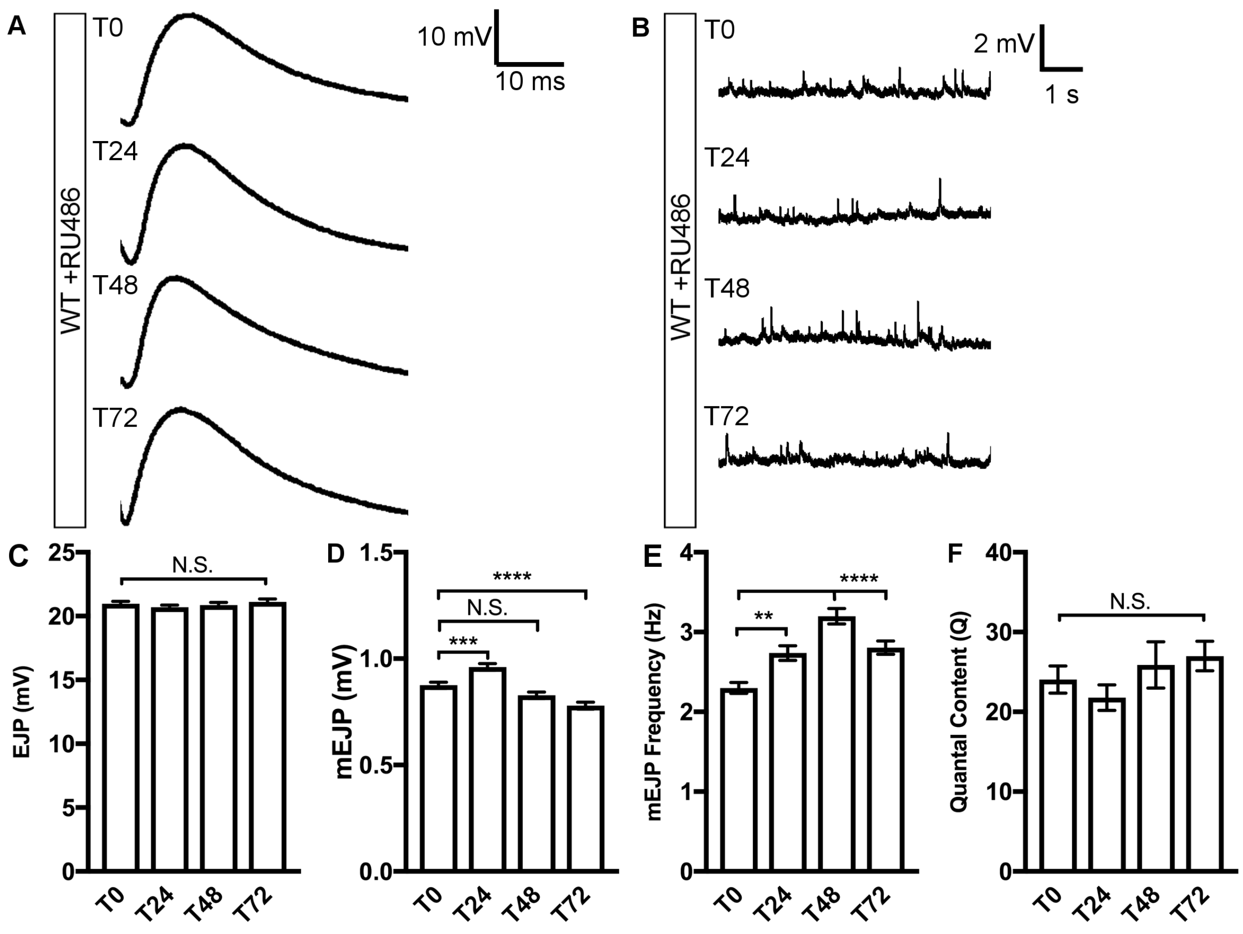

Supplemental Figure 5

RU486 alone does not lead to functional deficits

(A-B) Representative traces of EJPs **(A)** and mEJPs **(B)** from WT larvae raised on RU486. Time (T) represents time after exposure to the RU-486 (T_0, T_{24}, T_{48}, and T_{72}). **(C-F**) Quantification of EJP amplitudes **(C)** and mEJP amplitudes **(D)**, frequency **(E)**, and quantal content **(F)** N=7.

Error bars represent S.E.M. N.S.=p>0.05, **=p≤0.01, ***= p≤0.001, ****=p≤0.0001.

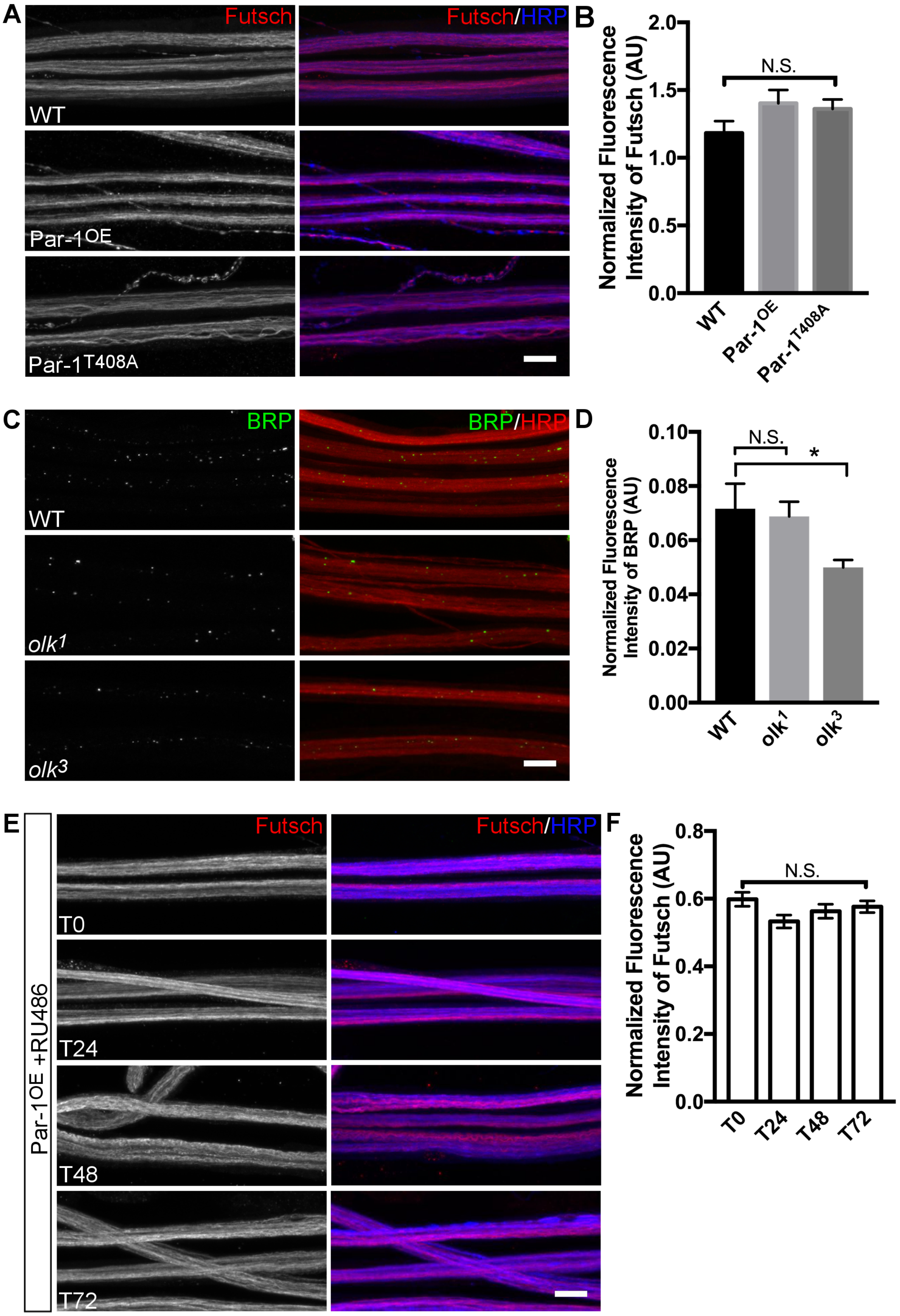
A
Futsch
Futsch/HRP
WT
Par-1OE
Par-1T408A
B
2.0
1.5
1.0
0.5
0.0
N.S.
Normalized Fluorescence Intensity of Futsch (AU)
WT
Par-1OE
Par-1T408A
C
BRP
BRP/HRP
WT
olk1
olk3
D
0.10
0.08
0.06
0.04
0.02
0.00
N.S.
*
Normalized Fluorescence Intensity of BRP (AU)
WT
olk1
olk3
E
Par-1OE +RU486
Futsch
Futsch/HRP
T0
T24
T48
T72
F
0.8
0.6
0.4
0.2
0.0
N.S.
Normalized Fluorescence Intensity of Futsch (AU)
T0
T24
T48
T72

Supplemental Figure 6

Futsch may not mediate the effects of elevated neuronal Par-1

(A) Representative confocal image stacks showing axon bundles from WT, Par-1^{OE} and Par-1^{T408A} third instar larvae stained with antibodies against Futsch (Red) and HRP (Blue). **(B)** Normalized Futsch fluorescent intensity (A.U.) within axon bundles is not significantly different. N=10. **(C)** Representative confocal stacks showing axons from WT, *olk*^{1}, and *olk*^{3} stained with antibodies against BRP (Green) and HRP (Red). **(D)** Mean fluorescence intensity of BRP from identical genotypes normalized to HRP. N=12. **(E)** Representative confocal stacks from larvae overexpressing Par-1 using GeneSwitch-ElavGal4 from T_0, T_{24}, T_{48}, and T_{72}. First column shows axons stained with an antibody against Futsch (Red) and second column with Merge of Futsch (Red) and HRP (Blue). **(F)** Mean Futsch fluorescence normalized to HRP levels within axons bundles. N=12. Error bars represent S.E.M. N.S.=$p>0.05$, *=$p\leq0.05$.

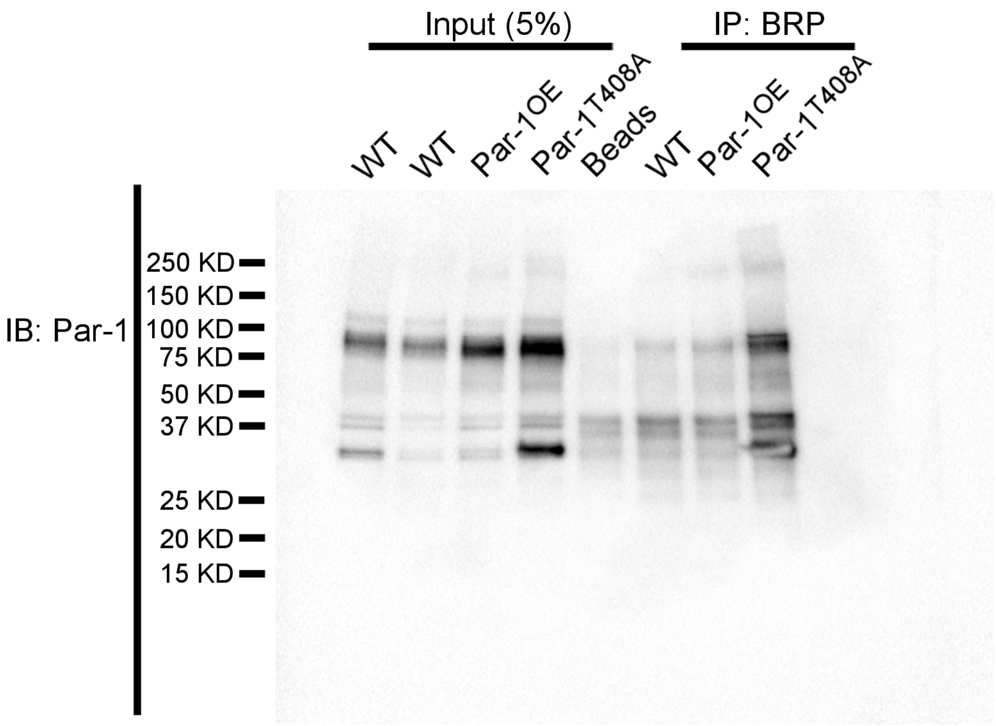

Supplemental Figure 7

Uncropped Western blots from Figure 7

Uncropped Western blots of proteins pulled down using the anti-BRP antibody and probed by anti-Par-1 antibody. Both the input and the IP were performed in the same blot and loaded on the same gel (different lanes).
